# Isolation and identification of *Acanthamoeba* from pond water of parks in a tropical and subtropical region in the Middle East, and its relation with physicochemical parameters

**DOI:** 10.1186/s12866-018-1301-x

**Published:** 2018-10-19

**Authors:** Shiva Ghaderifar, Ali Asghar Najafpoor, Hossein Zarrinfar, Habibollah Esmaily, Elham Hajialilo

**Affiliations:** 1 Student Research Committee, Mashhad University of Medical Sciences, Mashhad, Iran; 2 Social Determinants of Health Research Center, Mashhad University of Medical Sciences, Mashhad, Iran; 3 Global Center for Environmental Remediation, University of Newcastle, Callaghan, NSW 2308 Iran; 4 Allergy Research Center Mashhad, University of Medical Sciences, Mashhad, Iran; 5 Department of Epidemiology and Biostatistics, University of Medical Sciences, Mashhad, Iran; 6 Department of Medical Parasitology and Mycology, School of Medicine, Qazvin University of Medical Sciences, Qazvin, Iran

**Keywords:** Ponds water, Parks, *Acanthamoeba*, Tropical and subtropical region

## Abstract

**Background:**

Free-living amoeba (FLA) are wide-spread protozoa that are found in different environmental sources including water, soil, dust, hospital units and ventilation areas. These amoebas can act as opportunistic or non-opportunistic pathogens. Among FLAs, some genera such as *Acanthamoeba* are important because of their potential pathogenic ability in humans**.** The purpose of this study is to identify of *Acanthamoeba* isolated from pond water of parks in a tropical and subtropical region in the Middle East, and its relation with physicochemical parameters.From August to December 2015, 90 samples were collected from pond water of parks of 13 regions of Mashhad City. Physicochemical parameters were measured in situ. After filtering, the samples were cultured on Bacto-agar enriched with *Escherichia coli*. PCR analysis was conducted on the culture-positive samples, and then the PCR products were sequenced. Statistical analysis was performed by SPSS software and Fisher’s Exact and Mann-Whitney test.

**Results:**

Among the samples that were examined, 19 samples (21.1%) were positive for *Acanthamoeba*. The sequencing revealed that *Acanthamoeba* isolates belonged to T4 genotype of *Acanthamoeba*. There was no significant relationship between physicochemical parameters and the presence of *Acanthamoeba*.

**Conclusion:**

The prevalence of *Acanthamoeba* in pond water of parks was relatively high, but there was no significant relationship between physicochemical parameters and the presence of *Acanthamoeba*.

## Background

Free-living amoeba (FLA) are wide-spread protozoa that are found in different environmental sources including different types of water, soil, dust, vegetables, hospital units and ventilation areas [1]. This amoeba can act as opportunistic or non-opportunistic pathogens. Among FLAs, some genera such as *Acanthamoeba* are important because of their potential pathogenic ability in humans and animals [2]. Although some cases of *Acanthamoeba* infections have been reported in children with a healthy immune system, the amoeba can cause disease in at risk groups such as contact lens wearers, pregnant women, and diabetic patients; and immunocompromised patients including graft patients, AIDS and cancer patients [3].

*Acanthamoeba* can cause granulomatous amebic encephalitis (GAE), that is a severe infection of the central nervous system (CNS). Also, they can affect skin and lungs that may lead to death in high-risk individuals [4]. Amoebic keratitis infection can occur through the use of contaminated contact lenses with non-sterile water or through bathing or swimming in contaminated water [5]. Besides, FLAs serve as a carrier for different pathogenic bacteria such as *Legionella, Mycobacterium* and *Pseudomonas* [6]. *Acanthamoeba* has two stages in its life cycle: an active feeding trophozoite phase and dormant cyst phase. The cysts can occur in unfavorable conditions such as pH changes and dryness, lack of food and temperature variations [7].

*Acanthamoeba* has been divided into different genotypes (T1-T20) based on rRNA gene [8]. Among *Acanthamoeba* genotypes, the most prevalent type is T4 that cause disease in human [9].

Due to the risk of free-living amoeba in endangering human health, effective actions can be done to eliminate or control them by identification of contaminated sources. Water is the main reservoir for *Acanthamoeba*. Several studies worldwide have assayed different types of water to determine the presence of these amoebae in the water near human habitats [5]. As the city of Mashhad is a religious metropolis, and annually receives a large number of pilgrims from around the world, pay attention to this subject is more important.

The aim of this study is the isolation and identification of *Acanthamoeba* in pond water of parks in Mashhad city (a tropical and subtropical region in the Middle East) and their relations with the physicochemical parameters.

## Methods

### Sampling

Ninety samples were collected during August to December 2015 from pond water of parks in Mashhad city. The samples were taken from 15 different park’s ponds of 13 regions of Mashhad as listed in Table 1. In order to take water samples and sampling requirements standard method 2012 was utilized [10]. Physicochemical parameters including temperature, pH, total dissolved solids (TDS), electrical conductivity (EC) and turbidity were measured in situ by portable pH/EC/TDS/Temperature meter (model HANNA HI9813–5) and portable turbidity meter (model HACH 2100p). The samples were then transferred to the laboratory for subsequent microbiological analysis of *Acanthamoeba*.

**Table 1:** The sampling site and number of samples obtained from the pond’s water of parks

Region	Park’s name	Number of samples
1	Montazeri	6
2	Faramarz	6
3	Bahar	6
4	Basij	6
5	Omat	6
6	Narges	6
7	Salam	6
8	Koohsangi (2 ponds)	12
9	Setare	6
10	Shahab	6
11	Mellat (2 ponds)	12
12	Niayesh	6
13	Saee	6
Total		90

### Culture

About 500 ml aliquots of water samples were filtered through nitrocellulose membranes with 0.45 μm pore-size. Then, the filters were cultured on 1.5% Non-nutrient agar (NNA) medium of Bacto agar (Canada Quelab company) which was prepared with amoeba Page Saline and containing heat-killed *Escherichia coli* as food for the *Acanthamoeba*. Amoeba Page Saline consists of 2.5 mM NaCl, 1 mM KH_2_PO_4_, 0.5 mM Na_2_HPO_4_, 40 μm CaCl_2_-6H_2_O, and 20 μm MgSO_2_. 7H_2_O. The final pH of this solution reached about 6.9 with KOH [11].

The plates were incubated at room temperature, after at least 4 days each plate was observed daily under a light microscope to check for the FLA and were followed for 2 months. All of the plates were examined with a stereomicroscope for the trophozoite and cystic forms as positive plates. A piece of NNA medium agar which contained the amoebas in positive plates was chosen and placed on the solidified fresh NNA medium. The plates were incubated at 30 °C and were monitored daily for *Acanthamoeba* growth.

### Collecting *Acanthamoeba* from the medium

About 2000 μl of distilled water were added to the medium. Then, the surface and beneath of agar was scratched with lam edge to isolate *Acanthamoeba*. Trophozoites and cysts of *Acanthamoeba* were collected in *1.5 ml micro* centrifuge *tubes*. They were centrifuged for 10 min at 10000 rpm to remove additional agar.

### DNA extraction and PCR

Commercial Dyna Bio Genomic Mini Kit (Blood/Tissue DNA Extraction Mini Kit) was used to extract DNA according to the manufacturer’s protocol. Then, the DNA was stored at − 20 °C.

The PCR reaction was performed using JDP primers including JDP1 5′-GGCCCAGAT CGTTTACCGTGAA-3′ and JDP2 5′- TCTCACAAGCTGCTAGGGGAGTCA-3 [12].

These primers amplify an approximately 500 bp fragment. The amplification reaction mixture consisted 10 μl Master Mix (Taq DNA Polymerase, PCR buffer, dNTP, MgCl_2_) (Ampliqon, Denmark), 7 μl distilled water, 1.5 μl DNA and 1.5 μl the primers at a concentration of 5 mM. Total of 20 μl were mixed in a 0.2 *micro tube* and were placed in a Thermal Cycler device. Every PCR run contained a tube with distilled water instead of template DNA as a negative control. The cycles of PCR were set up as following: first denaturation step at 95 °C for 5 min and 35 cycles of denaturation at 95 °C for 30 s, annealing at 54 °C for 30 s and extension at 72 °C for 1 min and a final extension step at 72 °C for 5 min [12].

The PCR-products electrophoresis was done on 2% agarose gel, stained with the green viewer and visualized under blue light. The The PCR products were then sequenced (most using single sequencing) to confirm the amplified fragment and genotypic identification by using the same primers.

### Physicochemical parameters

To evaluate of presence *Acanthamoeba* in the ponds water, physicochemical parameters including temperature, pH, TDS, EC and turbidity were measured during the sampling.

### Statistical analysis

The data statistical analysis was performed by SPSS 16 software. Fisher’s Exact Test was used for comparison between different regions of parks. To determine the significance of normality, the Kolmogorov-Smirnov test was used. Also, Man-Whitney test was used for determination of relationship between physicochemical parameters and the presence of *Acanthamoeba.*

## Results

Among the 90 analyzed samples in this study, thirty one (34.44%) were positive for the FLA using culture along with direct microscopy. The trophozoite and cystic forms were usually visible after 1 week, although in some of the samples, they were visible after 1 month.

PCR analysis was conducted on the positive isolates and a specific 500 bp band was expectable on a agarose gel. Nineteen samples (21.1%) were positive for *Acanthamoeba* on PCR testing.

**Fig. 1:**
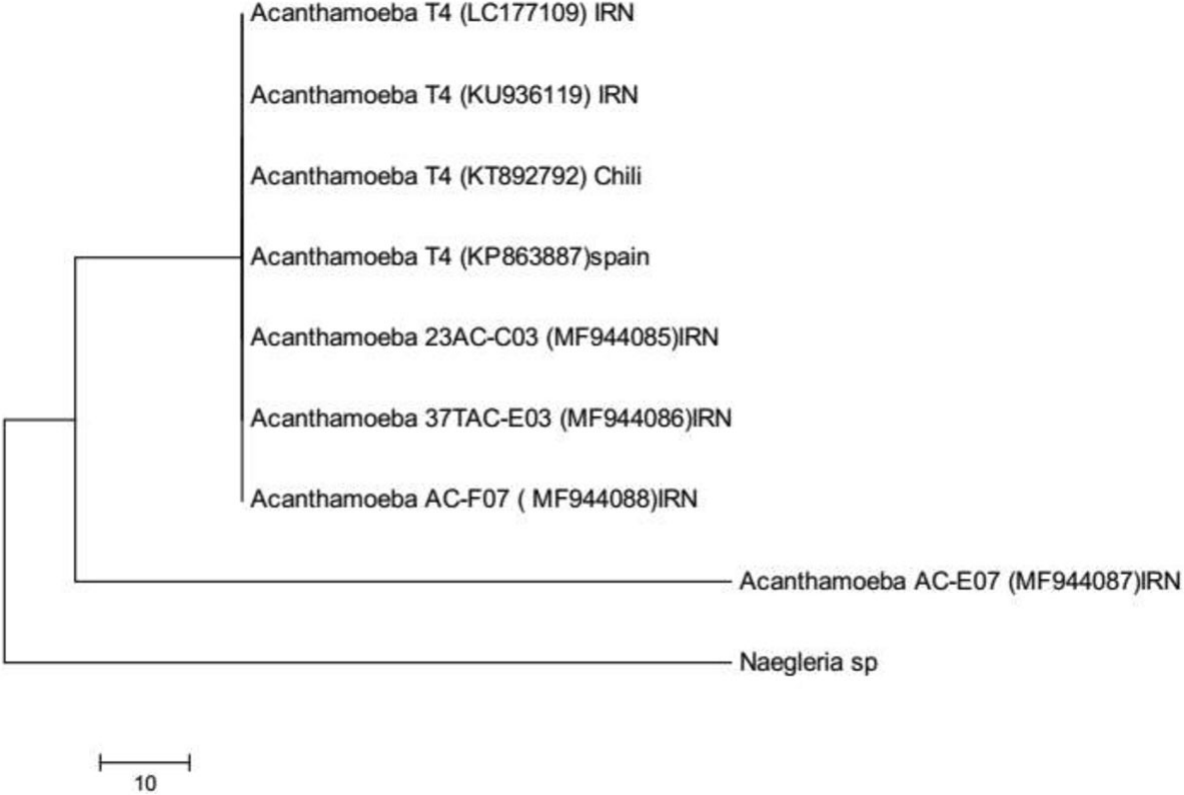
Phylogram derived from maximum likelihood analysis of the 18S rRNA gene sequence data for the *Acanthamoeba*

The 18S rRNA gene sequences of *Acanthamoeba* isolates were compared with *Acanthamoeba* sequences in the GenBank. The results revealed that the *Acanthamoeba* isolates belonged to T4 genotype. Figure 1 shows the phylogenetic tree based on sequenced isolates of T4. The genome sequences of the isolates were submitted to GenBank under the accession numbers MF944085, MF944086, MF944087, MF944088.

**Fig. 2:**
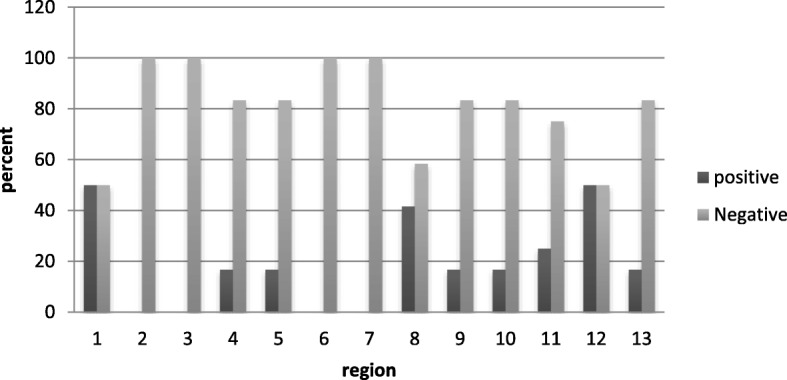
The frequency of *Acanthamoeba* distribution in pond water of parks in 13 regions of Mashhad city

Regions of 1 and 12 had the highest rate (percentage) of contamination for the *Acanthamoeba* isolates (Fig. 2). According to the Fisher’s Exact test, there wasn’t any relation between regions and the presence of *Acanthamoeba* (*p*-value, 0.32).

The Kolmogorov-Smirnov test results showed that none of the variables were significant in statistical level 0.05. Therefore Man-Whitney test was used for determination of relationship between physicochemical parameters and the presence of *Acanthamoeba.*

According to Table 2, there was no significant relationship between physicochemical parameters and the presence of *Acanthamoeba* (*p* > 0.05).

**Table 2:** Mean and standard deviation in positive and negative samples evaluated for *Acanthamoeba* isolates

Parameters	Positive/negative	Mean	Standard deviation	Median	Max	Min	Man-Whitney test result
Temperature	+ -	17.9 13.7	5.05 3.95	19.2 15	26.4 23.3	8 8	*P* = 0.06 Z = 1.8
pH	+ -	8.7 8.6	0.32 0.32	8.8 8.7	9.8 9.6	8.3 7.8	*P* = 0.45 Z = 0.74
EC	+ -	1.08 0.95	0.94 0.62	0.65 0.75	3.19 3.06	0.36 0.36	*P* = 0.37 Z = 0.88
TDS	+ -	496.3 626.6	132.7 341.8	464.5 525	739 1778	264 263	P = 0.06 Z = 1.8
Turbidity	+ -	2.35 2.17	1.84 1.96	2.06 1.41	7.22 7.66	0.47 0.47	*P* = 0.42 Z = 0.81

## Discussion

This study showed the prevalence of *Acanthamoeba* genus in ponds water of parks in Mashhad city as a tropical and subtropical region in the Middle East. Among the 90 samples that were examined in this study, 31 samples (34.44%) were positive for FLA using microscopic examination, and 19 samples (21.1%) were positive for *Acanthamoeba* using PCR method.

*Acanthamoeba* is abundant in environmental sources. Human contact with this potential pathogenic amoebic parasite during daily life and it can cause serious infections such as granulomatous encephalitis or amoebic keratitis [13].

Identification of *Acanthamoeba* is mainly based on cultivation on non-nutrient agar enriched with heat-killed *Escherichia coli*, and molecular methods [3]. The accuracy of culture along with direct microscopy is less than PCR method to identify of amoeba [14].

Di Filippo et al. study confirmed that culture method is not precise enough to detect of free-living Amoebae [8]. In the study, a total of 160 water samples was analyzed for FLA by PCR method. FLA were detected in 46 (28.7%) of the cultured water samples by microscopic examination, but using the PCR method on the culture, only 39 samples were positive. This may also be due to the concurrent presence of other amoebae in cultured samples, however, they cannot be correctly differentiated from each other using the direct microscopic examination of the *culture*-*positive* samples.

Based on Magnet’s study, PCR is more sensitive technique than direct microscopy of culture, but the use of both PCR and culture method is suggested for environmental water samples to gain more complete results of the real presence of *Acanthamoeba* [14].

The studies that examined the water samples collected from rivers and lakes showed a higher percentage of *Acanthamoeba* than our study [15]. The reason for decreasing the percentage of *Acanthamoeba* in the current study could be related to the replacing ponds water of parks with fresh water and destroying the biofilms. Because biofilms can accumulate all kinds of the microorganism providing sufficient requirement for the FLA [16]. Some bacteria can survive inside FLA, and they can multiply in FLA such as *Legionella and Mycobacterium* [17]*.* Scheikl revealed that *Legionella* spp. Co-Occurred with *Acanthamoeba* spp. [18]. Another study that was done by Ovrutsky showed that *Nontuberculous Mycobacteria* could grow in *Acanthamoeba* as parasites or as endosymbionts [19].

The sequencing analysis revealed that most of the *Acanthamoeba* isolates in this study belonged to T4. It is important to mention that T4 genotype is the most prevalent genotype have been reported or highly pathogenic among *Acanthamoeba* keratitis (AK) [20]. It indicates that pond water of parks in Mashhad city may be a source of acanthamoebic diseases in human.

In Iran, different *Acanthamoeba* was isolated from soil and water resources in different areas. The isolates belonged to different genotypes especially T4 [12]. A research was conducted by Armand et al. showed that the most prevalent genotype of *Acanthamoeba* in Shiraz water supplies is T4 [21].

Lass et al. collected 20 water samples from different sites in Poland. Of the 20 samples examined by PCR methods, 10 of them were positive for *Acanthamoeba*. The sequencing revealed that the isolates belonged to T4 genotype [15].

One of the limitations of this work was that we couldn’t do *direct sequencing for all of the positive samples*. Because the pond water of parks was polluted to many organisms, and so we couldn’t purify the PCR product perfectly and obtained some weak or *noisy sequences*.

In this study, the relationship of physicochemical parameters were evaluated on presence of *Acanthamoeba*. Few studies have investigated the effect of physicochemical parameters on the presence or absence of *Acanthamoeba.* However, to our knowledge, there wasn’t any study in this field in Iran yet.

The study showed that there was no significant relationship between physicochemical parameters and the presence or absence of *Acanthamoeba*. Tung et al. measured temperature, pH, and turbidity in their study. They revealed that the presence or absence of *Acanthamoeba* did not depend on these physicochemical parameters [22]. Another study that was done by Richard showed that there was no significant relationship between the existence of protozoa and the physicochemical parameters [23].

## Conclusion

The results showed the prevalence of *Acanthamoeba* in pond water of parks in Mashhad city was relatively high. Sequencing analysis revealed that *Acanthamoeba* isolates in this study were belonged to T4 genotypes. According to the statistical analysis of the results, there was no significant relationship between physicochemical parameters and the presence of *Acanthamoeba*. Due to contamination of the water and being exposed to individuals, especially children, the general advice is to *avoid* wearing contact lenses when swimming or showering, and also to create new strategies and control measures to reduce *Acanthamoeba*.

## Abbreviations

AK: Amebic keratitis
CNS: Central nervous system
EC: Electrical conductivity
FLA: Free living amoeba
GAE: Granulomatous amebic encephalitis
NNA: Non-nutrient agar
PCR: Polymerase chain reaction
TDS: Total dissolved solids
